# Descending necrotizing mediastinitis: etiopathogenesis, diagnosis, treatment and long-term consequences—a retrospective follow-up study

**DOI:** 10.1007/s00405-022-07769-x

**Published:** 2022-12-07

**Authors:** Thea Charlott Reuter, Valentina Korell, Jens Pfeiffer, Gerd Jürgen Ridder, Manuel Christoph Ketterer, Christoph Becker

**Affiliations:** 1 Department of Otorhinolaryngology, Medical Center, Faculty of Medicine, University of Freiburg, Killianstrasse 5, 79106 Freiburg, Germany; 2 HNO am Theater, Freiburg, Germany

**Keywords:** Deep neck infection, Mediastinitis, Collar mediastinotomy, Endo classification, Transcervical drainage, Quality of life

## Abstract

**Purpose:**

The primary aim of this retrospective study was to analyze the progression of descending necrotizing mediastinitis (DNM), evaluate the impact of comorbidities on complications and mortality and to observe long-term consequences of DNM on dysphagia and measurements quality of life. DNM is a serious infectious disease that requires multimodal treatment. Current literature varies in conclusions of risk factors, management and outcome of DNM. In addition, little is known about persisting effects on quality of life.

**Methods:**

Retrospective data analysis of 88 patients with DNM representing the largest single-center study. Recording data of patients and diseases as well as clinical progression from 1997 to 2018. Two questionnaires were sent to the participants to measure quality of life and to detect dysphagia.

**Results:**

88 patients were included. The most frequently found pathogen were *Streptococcus* spp. (52%). 75% of the patients underwent multiple surgeries, mean count of surgical procedures was 4.3 times. 84% received intensive care treatment. Median length of stay on the intensive care unit was 7 days. 51% had pre-existing comorbidities associated with reduced tissue oxygenation (e.g., diabetes). The most common complication was pleural effusion (45%). During the observation period, the mortality rate was 9%. 12 questionnaires could be evaluated. 67% of the participants were affected by dysphagia at the time of the survey.

**Conclusions:**

Descending necrotizing mediastinitis (DNM) is a severe disease requiring an immediate initiation of multimodal treatment. Although quality of life usually isn´t impaired permanently, dysphagia may often persist in patients after DNM.

## Introduction

Descending necrotizing mediastinitis (DNM) is a rare but rapidly progressive and often life-threatening form of mediastinitis. The infection originates from a head and neck source, mostly an odontogenic or oropharyngeal focus, extends via the deep fascial planes and descends into the mediastinum [1–3]. Formerly quite high mortality rates up to 40% decreased in recent years to an reported overall mortality rate of 17.5%, credited to the widespread use of antibiotics and improvement in diagnostic, surgical and interdisciplinary management [2, 4–6]. Chest pain, high fever and crackling on palpation are described symptoms of DNM, though these unspecific disorders do not necessarily be present. All the more expeditious recognition of DNM is crucially important to promptly initiate an appropriate surgical and wide-spread antibiotic treatment to reduce morbidity and mortality [4, 7–10]. In terms of potential risk factors to suffer from a DNM several pre-existing comorbidities, especially systemic conditions with a reduced tissue oxygenation, are described, such as diabetes mellitus, severe chronic nicotine and alcohol abuse, present tumor illness and chronic pulmonary and cardiovascular diseases [4, 11, 12]. In 1983 Estrera et al. established the following diagnostic criteria: (1) Clinical manifestations of severe infection; (2) Demonstration of characteristic radiographic findings; (3) Documentation of necrotizing mediastinal infection in operation and (4) Establishment of oropharyngeal/cervical infection with descending necrotizing mediastinitis relationship [1]. Computed tomography (CT) is an essential diagnostic medium which is used for clinical diagnosis and to identify the extent of mediastinal involvement and serves as a point of comparison for postoperative control [2, 5, 13, 14]. Microbiological culture usually reveals aerobic/anaerobic coinfections corresponding to its pharyngeal or odontogenic origins [4, 11]. Severe complications with high mortality of DNM are multiple organ dysfunction syndrome, sepsis or septic shock [2, 9, 15]. The prognostic impact of certain clinical predictors such as laboratory findings and patient specific data regarding outcome and intensity of the course of DNM is differently discussed [4, 11, 12]. In 2010 Ridder et al. published a study with 45 patients suffering from DNM treated at Department of Otorhinolaryngology Head and Neck Surgery, University of Freiburg between 1997 and 2008 [4]. Our retrospective study enlarges this collective of patients until December 31, 2018. With 88 patients the present study currently represents to our knowledge the biggest cohort treated at a single center. Our aim was not only to describe patient data and the individual course of DNM but also to characterize risk factors and outcome predictors. Moreover, we wanted to present data about long-term conditions, quality of life and dysphagia.

## Methods

This is an observational descriptive retrospective cohort study of 88 cases of DNM treated at the Department of Otorhinolaryngology and Head and Neck Surgery at the University Hospital of Freiburg Germany over a period of 21 years (January 1997–December 2018). Individual patients and diseases information as well as data of individual clinical progression of DNM were taken from in house software: symptoms, medical condition and clinical findings at the moment of presentation, laboratory, microbiological and radiographical studies were recorded. Beyond that we observed pre-existing comorbidities, source of infection, antibiotic and surgical treatment, interval between arising of symptoms and presentation, respectively, initiation of operation, duration of hospitalization and intensive care unit (ICU) stay and the number of operations performed. Subsequently we sent two questionnaires to all patients supposed to be alive: The Eating Assessment Tool 10 (EAT-10) was used to measure persistent swallowing difficulties and the Short Form (36) Health Survey (SF-36) was used to assess the actual state of health at the moment of interrogation. Diagnosis of DNM was established by clinical, radiographical and intraoperative findings. The diagnostic criteria of DNM defined by Estrera et al. were fulfilled [1]. The classification of Endo et al. was used to divide cases of DNM based on the anatomical extent in CT findings [16]. We excluded patients with mediastinitis from non-descending cause and also those with an iatrogenic etiology, e.g., postoperatively.

Unless stated otherwise, data are presented as mean (SD). Statistical analysis was done using R-based software (Jamovi, jamoviproject (2018), version 0.9.2.3.). Shapiro–Wilk test showed that data were not normally distributed, the Mann–Whitney *U* test was applied to continuous variables. To determine group differences for category variables Fisher’s exact test was applied. *P* ≤ 0.05 was considered statistically significant. To analyze the influencing factors on the dependent variable death we performed logistic regression analyzes. The study was approved by the ethics committee of the Albert-Ludwig-University of Freiburg (Votum Nr., 369/18, 07.02.2019).

## Results

### Demographic data

The mean age of the 88 patients included in the study was 54.7 years (median 55.5 years; youngest 3 years and oldest 84 years). 51 were male (57.9%) and 37 female (42.1%).

### Symptoms and source

At the time of presentation frequent symptoms were sore throat (*n* = 48, 54.6%), swelling and redness (*n* = 37, 42.1%), odynophagia (*n* = 30, 34.1%), dyspnea (*n* = 26, 29.6%) and dysphagia (*n* = 21, 23.9%). Furthermore, symptoms were fever (*n* = 17, 19.3%), neck pain and headache (*n* = 13, 14.8%), worsening of general condition and gnathospasm (respectively, *n* = 7, 7.9%), hoarseness (*n* = 6, 6.8%), foreign body sensation and upper back and/or shoulder pain (respectively, *n* = 5, 5.7%), sternal pain (*n* = 4, 4.6%) and stridor or stupor (*n* = 2, 2.3%). In most of the cases the etiology could not be reported with absolute certainty (*n* = 33, 37.5%). In 28 patients (31.8%), DNM originated from a previous infection, in 4, 4.6%, from post-radiogenic situation and one patient suffered from injection abscess. The time interval between onset of symptoms to admission was 3.53 days on average (median 3 days).

### Comorbidities

More than half of the patients (*n* = 45, 51.1%), suffered from pre-existing diseases with reduced tissue oxygenation: cardiac insufficiency (*n* = 28, 31.8%), diabetes mellitus (*n* = 16, 18.2%), respiratory insufficiency (*n* = 12, 13.6%), adiposities per magna (*n* = 6, 6.8%), post cervical radiation (*n* = 4, 4.6%) and peripheral arterial obstructive disease (*n* = 3, 3.4%). We calculated the individual Charlson Comorbidity Index of each patient, mean 2.9 points and median 2 points. 76.1% had ≥ 1 points. Chronic nicotine, alcohol and other substance abuse was detected in 31, 13 and 7 patients, respectively.

### Diagnostics

**Fig. 1:**
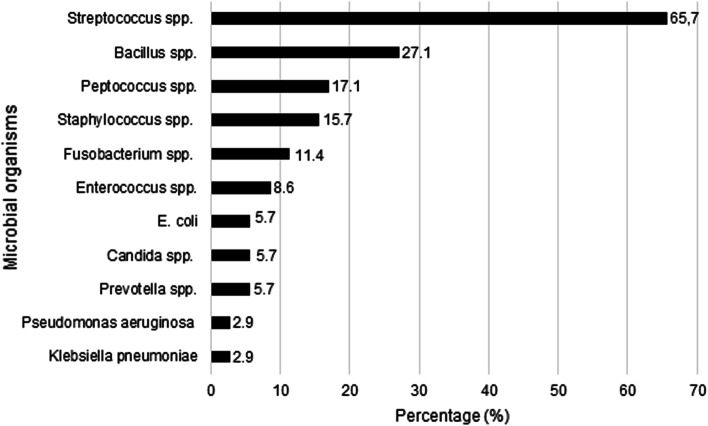
Distribution of microorganisms causing descending necrotizing mediastinitis

First step of the diagnostic pathway in any case was a thorough otolaryngologic examination. Amongst others laboratory findings on admission consisted of white blood cell (WBC) count and C-reactive protein (CRP). The WBC count ranged from 1.4 to 34.5 cells/mm^3^ (mean 14.9 cells/mm^3^, median 13.4 cells/mm^3^). CRP on admission ranged from 0 to 495 mg/L (mean 94.8 mg/L, median 39.6 mg/L). Microbiological examinations were obtained in 70 patients. In 50% of the microbiological examinations a mixed anaerobic and aerobic spectrum was detected. In 33 patients microbiological examination showed an anaerobic infection. In two patients, microbiological examination showed an exclusively aerobic infection. A detailed distribution of microorganisms is shown in Fig. 1. Depending on the patient`s condition B-mode ultrasound was performed. Another essential part of the diagnostic pathway were radiographic examinations. All of the 88 patients underwent imaging diagnostic. 86 patients received CT scans, at most 15 times (mean 3.14, median 2). 69 patients received CT scans in the follow-up. 16 patients (18.2%) underwent magnetic resonance imaging (MRI). In five cases MRI was performed multiple times. With the results of the radiographic imaging the conclusion of the localization of the DNM was achieved. Most affected site of the mediastinum was the upper part above the carina in 71 patients (80.7%) corresponding to Endo Type I. In 12 (13.6%), respectively, 10 (11.4%) patients the lower anterior (Endo Type IIA) or posterior (Endo Type IIB) mediastinum was involved as well.

### Therapy, trends, complications and mortality

**Fig. 2:**
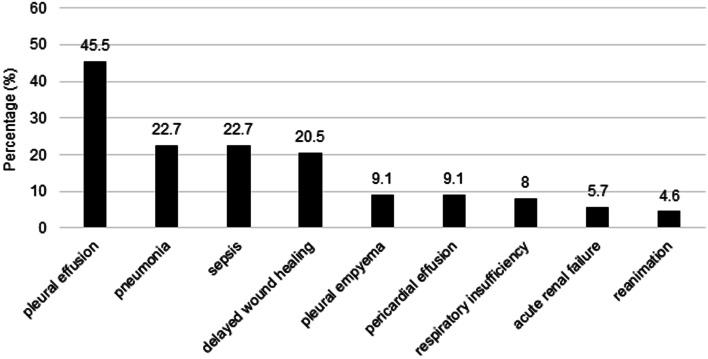
Complications during the treatment of descending necrotizing mediastinitis

Before presenting in our department 38 patients (43.1%) had already been treated with an antibiotic. At the latest at time of admission an empiric antibiotic therapy was started in all patients. Most frequently prescribed antibiotics were cephalosporine of the 2^nd^ or 3^rd^ generation (64 patients, 72.7%) often combined with metronidazole (61 patients, 69.3%) to cover the anaerobically spectrum of the infectious organisms. As soon as the results of the polymicrobial samples or the appropriate antibiograms were given a modification of the antibiotic treatment was performed if necessary. Indeed this was required in 50 patients of 88 (56.8%) in the course of the hospitalization. Complementing the antibiotic therapy a surgery was performed in 83 patients. 69 patients (83.1%) underwent a panendoscopy, 53 patients (63.7%) outer transcervical drainage, 48 patients (57.8%) collar mediastinotomy. Inner drainage of the abscess was executed in 31 patients (37.6%), tracheotomy in 29 patients (34.9%) and foreign bodies were extracted in five patients (6%). 15 patients were operated only a single time. 66 patients underwent multiple surgical procedures (mean 4.26, median 3, maximum 26). To ensure wound drainage, cervical incisions were sutured incompletely and with the help of large lumen tubes inserted in the wounds the situs was daily irrigated with antiseptic solutions. If required this took place in repeated sessions under general anesthesia. The mean duration of hospitalization was 25.5 days (median 18 days, maximum 139 days). 74 (84.1%) patients were admitted to an intensive care unit (ICU). The mean duration of ICU stay was 13.3 days (median 7 days, maximum 110 days). During the treatment of DNM, a total of 64 patients (72.7%) developed complications (Fig. 2). 8 of our 88 patients with DNM died, thus the mortality rate was 9.1%. Death occurred meanly after 11.4 days past admission (median 2.5).

### Group differences and influencing factors on mortality

Analysis of relevant group differences between survivors (*n* = 80) and deceased (*n* = 8) was conducted. This revealed that those patients who died were distinctly older (mean age 67.1 years in patients who died, mean age 53.5 in patients who survived DNM; *p* = 0.04) and had higher CRP levels on admission (mean CRP 239.7 mg/L in patients who died, mean CRP 80.3 mg/L in patients who survived DNM; *p* = 0.05). No significant differences between survivors and deceased were found in the WBC count on admission, extent of involved mediastinum and interval between onset of symptoms and admission. In addition, pre-existing comorbidities, diabetes mellitus, nicotine abuse and antibiotic therapy before admission were not relevantly different between the two groups. Of all the observed factors CRP level on admission (*p* = 0.01) and greater age of the patients (*p* = 0.05) showed a significant impact on the mortality of our patients.

### Quality of life and dysphagia in the long-term

Of all the patients being sent the questionnaires only 12 patients answered. The mean interval between DNM and interrogation was 106.7 months (median 107.5 months). The mean age of the patients at the time of interrogation was 53.2 years (median 60.5 years; minimum 12 years and maximum 84 years). Concerning the EAT-10 questionnaire helping to detect swallowing restrictions and dysphagia 8 of the 12 patients reached a score ≥ 3 points, indicating abnormal high values. The highest score was 22 points (mean 8.2 points, median 5.5 points). One patient declared not to have any problems at all (0 points). Highest score was assessed to the statement “I cough when I eat” (mean 1.5 points, median 1.5 points), whereas odynophagia was weighted least (mean 0.3 points, median 0 points). The SF-36 questionnaire represents the health status during the last 4 weeks at the time of filling in. Comparative values are represented by average data of a general population. Our patients´ answers were partially incomplete which made it impossible to receive complete records of all of the 12 patients. Health-related quality of life is sectioned into physical and mental health status. SF-36 revealed, that the physical health summary is above average in 5 patients and below average in 4 patients. The mental health score lays about average in 7 patients and below average in 3 patients.

## Discussion

First described in 1938 by Pearson et al. DNM remains a serious, aggressive disease often associated with fatal outcome [3, 5, 17, 18]. Even though it is a rare disease DNM should not be underestimated. It can still lead to sepsis and death, while once high mortality rates from 49% in 1938 decreased during the last years to 17.5% described in a review by Prado-Calleros in 2016 as diagnostics and multimodal therapy concepts improved [4, 5, 19–21]. Deep neck infections, DNI, develop from odontogenic or oropharyngeal infections and rapidly spread via deep fascial planes downward into mediastinum resulting in DNM [2, 11, 22, 23]. Based on its pattern of spreading Endo et al. developed a classification of CT findings: (I) focal form: localized to the upper mediastinal space above the carina. (II) Diffuse form reaches out below the tracheal bifurcation and is subdivided into (IIA): lower anterior mediastinum and (IIB): lower posterior mediastinum [16].

Common literature agrees that a diagnosis at early stage of DNM as well as a prompt initiation of appropriate medical and radical surgical treatment are imperative [2, 4, 9, 11, 17, 24–29].

However, an immediate recognition of DNM can be challenging due to the following reasons: DNM is a rare disease not only ENT experts and general surgeons but also family doctors, pediatricians and general practitioners should keep in mind. Though only 2–3% of deep space neck infections develop to more serious infections, such as mediastinitis, one should always be aware of such a severe course as it can progress very fast [7, 30, 31]. Furthermore, it can affect all age groups as the results of our investigation, age range 3–84 years, show in concordance with the literature. In doubt the clinical picture is sufficient to suspect DNM and the patient should promptly be admitted to a hospital with ENT specialist for thorough examination of the pharynx and larynx including fiberoptic transnasal endoscopy and efficient initiation of imaging diagnostics [4, 32]. Moreover, symptoms may not be distinct. The infection is often clinically silent especially at the beginning and may be veiled by analgesics delaying the diagnosis. Therefore, a clear association between symptom, severity and extent of the disease is difficult [4, 12, 13, 32, 33].

In our study most common clinical symptoms, pain, swelling and odynophagia, at the time of presentation are related to common oropharyngeal infections and DNIs. However, disorders correlating explicitly with mediastinitis such as chest pain and mediastinal emphysema are less frequent in our patients [15, 27, 33, 34].

The clear relation between infections of an odontogenic or pharyngeal source and the development of DNM is undoubted [9, 17, 35]. The anatomic continuity of the posterior pharyngeal, parapharyngeal and submandibular spaces with the mediastinum explains this smooth transition. Once an infection enters one of these spaces a spreading downward is promoted because of gravity, respiration, intrathoracic negative pressure and absence of barriers in fascial planes [4, 35, 36]. The so-called danger space lies posterior to the alar fascia, runs from skull base to diaphragm and allows even a contralateral spread of the infection [9].

Also, in our patient group previous infection of odontogenic or pharyngeal origin is often mentioned. Four patients even reported a post-radiogenic situation of the neck. This underlines the importance of a diligent execution of medical history as these patients are more likely to suffer from wound healing disorders [37].

Several predisposing risk factors are well-recognized referring to patients with DNM, such as diabetes mellitus, poor dental or oral hygiene, immunosuppression, renal and liver failure, high blood pressure and recent steroids. Moreover, chronic nicotine, alcohol and IV drug abuse happens to appear frequently in patients with DNM [9, 11, 12, 15, 38–40]. The description that patients with DNM suffer significantly more often from comorbidities compared to patients with DNI underlines the high impact of these pre-existing risk factors to develop DNM [10, 12]. Our analyses turned the attention especially to comorbidities with a reduced tissue oxygenation as these expedite the development of DNM [4]. More than half of our patients suffered from pre-existing diseases associated with impaired tissue oxygenation, e.g., cardiac or respiratory insufficiency, diabetes mellitus, adiposities per magna and post cervical radiation. This coincides with the observations of Kocher et al. [2]. Nonetheless also young, healthy patients with no medical history can suffer from DNM [41–43].

The Charlson Comorbidity Index, CCI, is a well-approved statistical test to predict the mortality of patients based on underlying comorbidities and has widely been used since its first description in 1987. Patients are divided in four groups (0 points, 1–2 points, 3–4 points and 5 points) correspondent to an increasing mortality risk. We used the age adjusted variant [44, 45]. Only 23.9% of our patients had an index of 0 points. Park et al. observed a CCI ≥ 1 in 54.1% in a group of 135 patients with DNI [46]. The results of CCI in our study underlines even more the role of comorbidities in patients with DNM and indicates, that patients with pre-existing reduced health conditions may have a higher risk to develop DNM.

The polymicrobiological nature of DNM as often described in diverse studies makes sense considering its origin as an oropharyngeal infection that once it has penetrated the mucosa spreads downward to the mediastinum [2, 33]. Likewise, Palma et al. half of our patients had mixed anaerobic and aerobic infections [11]. Nonetheless no result regarding extensive microbiological examinations was obtained in 18 of our patients. This lack of microbiological results was observed as well by other authors. It might be due to the fact that quite a high number of patients, e.g., in our study 43.1%, has already been treated with antibiotics before microbiological examinations was obtained [4, 12, 32].

In more than three-quarter of our patients the upper part of mediastinum above the carina was affected corresponding to Endo Type I. CT scans are an early part of the diagnostic pathway and crucial in recognizing DNM before it spreads even deeper. Radiation exposure should be accepted in case of deep neck infection or just its clinical suspicion to exclude DNM yet at the beginning. Freitas et al. even suggests an algorithm with serial CT scans every 24–48 h to assess disease progression [32]. In line with that also most of our patients received CT scans in the follow-up to monitor the course of DNM and if applicable to act immediately. The expressiveness of CT scans after repeated surgical interventions with changed tissue textures can be hindered. Moreover, laboratory findings as WBC or CRP levels can be indistinct, e.g., if they do not decrease conspicuously. In case of doubt, we, therefore, favor a liberal decision to revision surgery.

The surgical drainage of the affected parts of mediastinum is without any doubt mandatory. However, there is still some controversy about the optimal approach. A proper approach should thoughtful be chosen according to patients condition, the extent of the disease and also the surgeons experience [2]. Responsible for the mortality of DNM is not only a delayed diagnosis but as well an inappropriate drainage of the mediastinum. Therefore, the latter should also carefully be focused on [11]. As most of our patients suffer from DNM Endo Type I, where infection is located in the upper part of the mediastinum, we are of the opinion that in these cases a sole transcervical drainage is sufficient for surgical debridement and necrotic tissue removal. A highly aggressive surgical treatment irrespective of the extent of DNM as formerly advocated by many authors can also implicate disadvantages and may go along with a higher complication rate [6, 13, 28, 33, 36]. In case of an advanced stage of DNM, Endo Type II, we also certainly support a thoracic approach via thoracotomy in combination with transcervical debridement for drainage of the upper and lower parts of mediastinum. Most accomplished is a posterolateral thoracotomy on the more affected side [5, 18]. As healing of DNM may often be protracted and revision surgery is commonly needed we usually perform incomplete closure and insertion of large tubes in the wounds combined with daily irrigation with antiseptic solutions.

The infectious process of DNM can frequently lead to pharyngolaryngeal edema and consequently cause dyspnea. We recommend in these cases of foreseeable difficult intubation an awake or at least video assisted intubation if necessary in preparedness of coniotomy. If upper airway is compromised or patients seem to need a long-term treatment because of severity of DNM we approve tracheotomy. Nevertheless, we refuse to overhasty decisions for routine tracheotomy in patients with DNM as spreading of cervical infection may occur [2, 5].

Amongst our patients mortality was significantly influenced by higher age and higher CRP-levels. Although not relevant to mortality in the present survey we do think other factors as comorbidities, extent of involved mediastinal site and duration of discomfort until treatment still have an impact on progress and outcome of DNM. Therefore, the overall impression of the patient must not be disregarded.

We are confronted with a modest number of questionnaires sent back by our patients who were also treated at ICU. Besides we face a heterogeneous distribution of time passed, since patients suffered from DNM. In addition, Gerth et al. report a loss of patients in follow-up controls in their review regarding changes in health-related quality of life after ICU [47]. Amongst others a reason to not sending back the questionnaire might be the long period of time of more than 20 years included. Patients suffering from DNM years ago not only became essentially older as well as their health condition presumably impaired and, therefore, precluded answering and sending back the questionnaires. Basically the SF-36 we used is well-approved and the most employed questionnaire to evaluate quality of life after critical illness. Despite the quantity our findings implicate a tendency that patients post DNM rather suffer from impaired physical than mental health in long-term. This is concomitant with the results of Gerth et al. [47]. Kramer et al. indeed reported that physical-functionary health-related quality-of-life subscales remain depressed even years after the acute illness in patients after ICU [48].

Our findings implicate that persisting dysphagia after DNM actually occurs hence two-thirds of our patients had above average high values in EAT-10 questionnaire. This corresponds to the observation that dysphagia is well-recognized as a late complication of DNM by several authors. We likewise subscribe to the advice of comprehensive long-term physiotherapy and logopedic support in patients with DNM not only to prevent aspirations but also to improve their quality of life [8, 17, 40].

## Conclusions

Despite great advances in diagnostics and treatment of DNM it remains a severe disease that requires a prompt, strenuous and multidisciplinary approach to prevent fatal outcomes. Particularly high CRP levels and old age have a remarkable influence on mortality. Nonetheless comorbidities, e.g., associated with impaired tissue oxygenation should not be disregarded. Even though acuteness of DNM is conquered long-term influence as dysphagia and in some cases declined quality of life may persist.
